# Effect of Blood Flow on Cardiac Morphogenesis and Formation of Congenital Heart Defects

**DOI:** 10.3390/jcdd9090303

**Published:** 2022-09-08

**Authors:** Fernando Trinidad, Floyd Rubonal, Ignacio Rodriguez de Castro, Ida Pirzadeh, Rabin Gerrah, Arash Kheradvar, Sandra Rugonyi

**Affiliations:** 1 Biomedical Engineering Department, University of California, Irvine, CA 92697, USA; 2 Biomedical Engineering Department, Oregon Health & Science University, Portland, OR 97239, USA; 3 Department of Cardiothoracic Surgery, Stanford University, Stanford, CA 94305, USA

**Keywords:** cardiac hemodynamics, heart development, mechanotransduction, biofluid mechanics, congenital heart disease

## Abstract

Congenital heart disease (CHD) affects about 1 in 100 newborns and its causes are multifactorial. In the embryo, blood flow within the heart and vasculature is essential for proper heart development, with abnormal blood flow leading to CHD. Here, we discuss how blood flow (hemodynamics) affects heart development from embryonic to fetal stages, and how abnormal blood flow solely can lead to CHD. We emphasize studies performed using avian models of heart development, because those models allow for hemodynamic interventions, in vivo imaging, and follow up, while they closely recapitulate heart defects observed in humans. We conclude with recommendations on investigations that must be performed to bridge the gaps in understanding how blood flow alone, or together with other factors, contributes to CHD.

## 1. Introduction

During embryonic and fetal development, the human heart grows while it gradually transforms from a valveless tubular structure into a four-chambered organ with valves [1,2]. As soon as a primitive tubular heart is formed, the heart begins beating and circulating blood. Subsequent interactions between heart tissues and blood flow shape the heart’s morphology and function [3,4,5]. The sensitivity of cardiac cells to blood flow acts as a feedback mechanism that guides the development of the heart, allowing the growing heart to adapt to the changing environmental and embryonic conditions. Thus, heart morphogenesis depends on a highly orchestrated interchange between genetic and epigenetic processes that reciprocally interact with blood flow. When abnormal blood flow disrupts the normal interactions between genetic and epigenetic programs, cardiac malformation in the form of congenital heart disease (CHD) may take place. The interaction between blood flow and cardiac morphology extends beyond the early stages of cardiac development, and it could be responsible for some heart diseases acquired later in life.

CHD manifests as one or more defects in a newborn’s heart. The etiology of CHD is multifactorial, with several factors contributing to its risks [1,6,7]. Well known and relatively well-studied factors include embryonic genetic and epigenetic anomalies (e.g., the 22q11.2 deletion associated with DiGeorge syndrome), some maternal habits (e.g., smoking, alcohol, and recreational drug consumption), and maternal conditions (e.g., undernutrition, diabetes, infectious diseases). In the absence of genetic and epigenetic anomalies and/or exposures to teratogens, abnormal blood flow can lead to a variety of CHDs [8,9,10]. Regardless of whether abnormal blood flow is the primary cause of CHD or a secondary cause, hemodynamics affects molecular signaling during embryonic and fetal stages of heart development, regulates cardiac morphology, and impacts heart function. Elucidating the role that blood flow plays in heart development and CHD formation is crucial to better understand cues that lead to CHD and to devise strategies and interventions to prevent heart defects or treat patients with CHD.

In what follows, we first provide a brief overview of heart development. We then discuss the current state-of-the-art studies aiming at elucidating the mechanisms through which blood flow regulates cardiac development and how abnormal flow may lead to CHD and detrimentally affect tissue formation. We conclude with an overview of some of the achievements and challenges in the journey to understand the relationship between blood flow and CHD.

## 2. Heart Development

**Figure 1:**
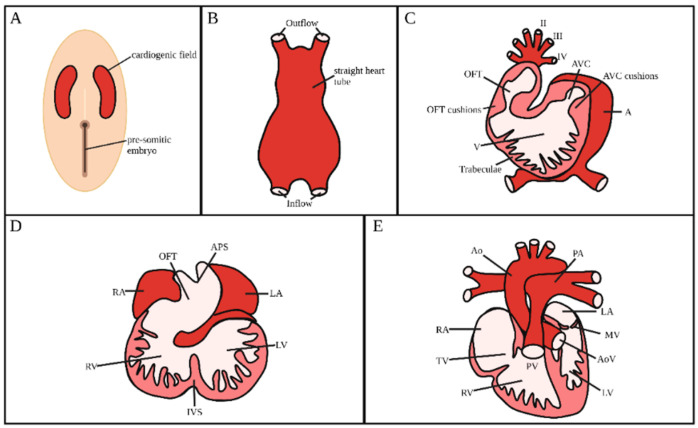
Developmental landmarks of cardiac development. (**A**,**B**) Cells located within the cardiogenic field migrate towards the midline to form a straight heart tube. (**C**) The heart begins to loop and can be compartmentalized into the primitive atria, atrioventricular canal, primitive ventricle, and outflow tract. Concurrent with heart looping, sheet-like projections called trabeculae begin to form. (**D**) Once heart looping is complete, septation (division) initiates within the ventricles, atria, and outflow tract. The outflow tract continues to rotate to align the future aorta and pulmonary artery with their respective ventricle. (**E**) The normal mature heart consists of two atria, two ventricles, four valves, as well as aortic and pulmonary outlets. OFT: outflow tract; AVC: atrioventricular canal; V: primitive ventricle; A: primitive atria; RA: right atria; LA: left atria; RV: right ventricle; LV: left ventricle; APS: aorticopulmonary septum; IVS: interventricular septum; Ao: aorta; PA: pulmonary artery; MV: mitral valve; AoV: aortic valve; TV: tricuspid valve; PV; pulmonary valve; II, III, IV: aortic arches II, III, IV. Created with BioRender.com (accessed on 24 July 2022).

Throughout embryogenesis, the forming heart experiences major morphological transformations by remodeling a field of cardiac progenitor cells into a four-chambered organ consisting of two atria, two ventricles, and four heart valves. Achieving this transformation requires cellular processes, such as migration, proliferation, apoptosis, and differentiation, as well as cellular signaling, to proceed in a precise and coordinated manner [1]. Although many aspects of cardiac development remain elusive, the detailed mechanisms by which this highly orchestrated coordination of events occurs are emerging. Major developmental landmarks in the developing embryonic heart include heart tube formation, looping, cushion formation and maturation, and septation. Figure 1 provides an overview of heart development. If any of these processes go awry at any time point during heart development, a variety of congenital heart defects may manifest.

### 2.1. Heart Tube Formation

During early stages of embryonic development, the mesoderm divides into the somatic and splanchnic mesoderm. A subset of the splanchnic mesoderm differentiates into a layer of myocardial progenitor cells, generating a bilateral field of cardiac primordia [11]. A population of these cells ultimately delaminates from the cardiogenic field and reside between the mesodermal and endodermal layers to form a plexus of endodermal precursor cells, which then coalesce to form bilaterally paired tubes (Figure 1A) [2,12]. As the foregut pocket is generated from invagination of the endoderm, the heart field and endodermal precursor cells simultaneously fold and fuse at the ventral midline [11,13,14,15]. During the migration of the mesoderm, myocardial progenitor cells concomitantly produce cardiac jelly, a cell-free, gelatinous substance that provides a separation between the prospective myocardium and endocardium [16]. This series of events lead to a primitive heart tube (Figure 1B) consisting of an endocardial layer enveloped by a myocardial mantle with both layers separated by cardiac jelly. Immediately after formation of the tubular heart, myocardial cells begin to cyclically contract and soon establish blood circulation within the embryo.

### 2.2. Heart Looping

After the formation of the heart tube, cardiac looping transforms the primitive linear, straight heart tube into an S-shaped tube (Figure 1C). Asymmetrically expressed factors within the left-right axis govern the direction in which the heart tube loops, creating a C-shaped heart tube with a prominent outer curvature and a narrow inner curvature [2,17,18]. Concurrently with heart looping, progenitor cells from the second heart field are added to the outflow and inflow segments of the tube [19,20]. Migration of these cells into the heart tube elongates the tube, enabling it to properly rotate and loop into an S-shape. At the end of looping, the heart tube, from inflow to outflow, consists of the primitive atrium, atrioventricular canal (AVC), primitive ventricle, and the outflow tract (OFT) [2]. The OFT (also referred to as conotruncus) consists of a proximal portion, the *conus*, and a distal portion, the *truncus*. After looping is complete, septation starts to transform the tubular heart into a four-chamber heart.

### 2.3. Formation and Maturation of Endocardial Cushions

**Figure 2:**
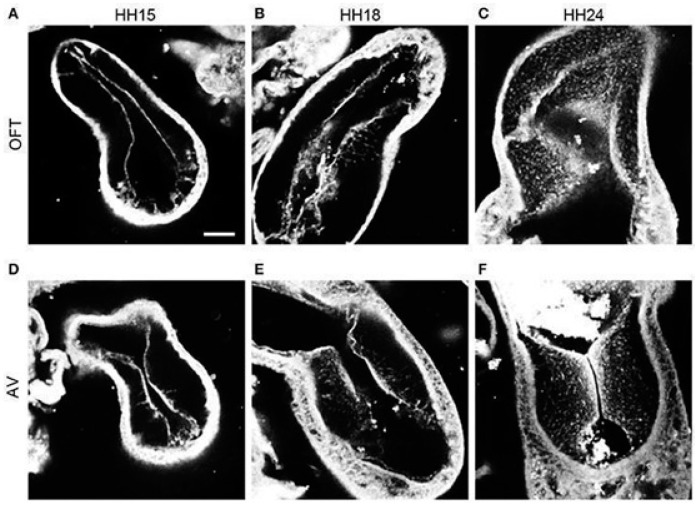
Progression of endothelial-mesenchymal transition (Endo-MT) in chicken endocardial cushions. Confocal fluorescent images were obtained at 20× from whole-mount embryo hearts stained with phalloidin to contrast cell content. (**A**–**C**) Outflow tract (OFT) cushions at progressive early developmental stages (HH15, HH18 and HH24, respectively). (**D**–**F**) Atrioventricular canal (AVC) cushions at progressive developmental stages (HH15, HH18, and HH24, respectively). Scale bar = 100 µm. Reproduced from [27], under Creative Common License.

During tubular heart stages, endocardial cushions form in the OFT and AVC of the developing heart (see Figure 2), providing the initial structural framework for formation of valve leaflets and septa. Initially, endocardial cushions are localized paired swellings of acellular cardiac jelly that are then invaded by cells [21]. While the AVC has a pair of cushions, in the OFT the conus and the truncus have separate cushions. Prior to the maturation of the cushions into leaflets and septa, they serve as ‘primitive valves’ to establish unidirectional flow [22].

Cushion maturation differs based on their location. Cushions located at the AVC and the OFT conus acquire mesenchyme (i.e., cells) through a process called endocardial-to-mesenchymal transition (Endo-MT). During Endo-MT, endocardial cells delaminate from the endocardium, acquire a mesenchymal phenotype, and invade the cardiac jelly [23,24]. These mesenchymal cells then proliferate to further populate the cushions. Subsequently, the cushions remodel to become valve leaflets. During cushion remodeling, the mesenchymal cells undergo condensation, a process characterized by increased cellular density in a particular area. In this case, condensation occurs just beneath the endocardium and is important for the formation of mature thin valve leaflets [25,26]. Conversely, the OFT truncal cushions acquire mesenchyme from Endo-MT but also from an extra-cardiac origin, as cardiac neural crest cells migrate and differentiate into the cushions [24]. The truncal cushions fuse to become the aorticopulmonary septum (APS) that separates the aorta from the pulmonary artery.

### 2.4. Trabeculation and Ventricular Formation

Concurrent with heart looping and endocardial cushion formation, the myocardium located within the outer curvature of the primitive ventricle starts to expand inwards and form trabeculae, which are spongy sheet-like projections (Figure 1C) [28]. Trabeculae formation increases the heart’s endocardial surface area, enhancing nutrient exchange. Additionally, the trabeculae augment ventricular contractile force.

As the embryo grows, the ventricles mature and remodel to (1) accommodate a larger volume of blood entering the heart, and to (2) supply sufficient blood flow to the developing embryo. Several morphological and functional changes must occur to achieve this. One of these changes is the initial thickening of compact myocardium (the main ventricular wall that excludes the trabeculae) through cellular proliferation. This process is followed by compaction of the trabeculae within the ventricular wall [29,30]. Eventually, compact myocardium will constitute most of the ventricular walls, with cells and fibers of the compact myocardium aligned in a spiral pattern that reflects the twisting motion of the ventricles during contraction [29]. Concurrently, the ventricular cavities enlarge to accommodate the increase in blood volume. All these processes must precisely occur in tandem to develop normal functioning ventricles.

### 2.5. Atrial and Ventricular Septation

Once the heart completes looping and the endocardial cushions are formed, septation (division) of the heart compartmentalizes atrial and ventricular chambers along with the aortic and pulmonary outlets (Figure 1D). The atrial and ventricular chambers are first septated through fusion of the AVC cushions [24]. Atrial septation begins with a muscular septum protruding from the atrial roof into the AVC. In parallel, a muscular interventricular septum protrudes from the deepest part of the outer curvature and expands to fuse with the AVC cushions to divide the ventricles.

### 2.6. Alignment and Septation of the Outflow Tract

Before septation, the OFT is a single conduit through which blood exits the tubular heart. During OFT septation, fusion of the truncal cushions leads to the formation of the aorticopulmonary septum (APS) that separates the pulmonary and aortic arteries, and after division of the OFT, truncal cushions are remodeled into aortic and pulmonary valves. The conal cushions contribute only to a small portion of the interventricular septum [2].

Remodeling and septation of the OFT into the great vessels happen through two distinct phases: (1) the OFT must correctly elongate and rotate to ensure that the future aorta and pulmonary trunk are aligned with their respective ventricle, and (2) the OFT must properly septate into the aorta and pulmonary artery. Phase 1 occurs at the end of heart tube looping (see Section 2.2). Once proper rotation and alignment is complete, the common OFT septates to generate the arterial vessels. As described earlier, prior to septation, neural crest cells migrate into the heart and populate the truncal cushions, which then fuse to form the APS. This is the initial division into the aorta and pulmonary trunk [24,31]. The APS then begins to expand and spiral into the conus. As the APS approaches the conus, the conal cushions close the septum in a ‘zipper-like’ fashion, completing the septation process [2,24,31]. At the end of septation, the heart is fully formed (Figure 1E), but it continues to mature during subsequent developmental stages.

### 2.7. Contributon of Extra-Cardiac Cells

Proper morphogenesis of the heart also depends on extra-cardiac cells that are outside of the initial cardiogenic field. Neural crest cells migrate towards the heart, contributing smooth muscle cells to the developing pharyngeal arch arteries, and then continue to migrate into the heart to contribute mesenchyme to the OFT. Recently, it has been suggested that neural crest cells may also contribute to the myocardium [32]. Moreover, second heart field cells also migrate into the heart and contribute to both the inflow and outflow poles. A subset of the second heart field termed the ‘secondary’ heart field contribute to the myocardium of the OFT and is essential for OFT septation and rotation. Likewise, cells from the proepicardium, a transient organ located at the base of the venous pole of the developing heart, migrate to the AVC to establish the epicardium. These cells proliferate to completely envelop the heart. A subset of the nascent epicardial cells will undergo an epithelial-to-mesenchymal (EMT) transition to invade the underlying myocardium and subsequently differentiate into (1) fibroblasts that contribute to extracellular matrix (ECM) deposition within the developing heart, and (2) vascular smooth muscle cells that ultimately form the coronary vasculature [33].

### 2.8. Conduction System

Concurrent with formation of the ventricles and cushions, development of the His–Purkinje system (HSP) takes place. The HSP is a set of tissues that coordinate the activation of myocardial cells enabling heart contraction. The HSP is composed of the Bundle of His, which receives signals from the atrioventricular (AV) node and propagates it down the Purkinje fibers to initiate ventricular contraction of cardiomyocytes [34]. During the initial stages of embryonic development, cardiac impulses propagate linearly from the sinus venosus at the inlet of the tubular heart toward the OFT. Activation speed is spatially non-uniform throughout the looping stages, with faster conducting segments located within the early atria and ventricles. Although impulse propagation speed increases during development, the signal follows the direction of blood flow. This means that activation initiates in the AV junction, propagates along the primitive left ventricle toward the ventricular apex, and ends by going through the primitive right ventricle and to the OFT. This directionality is referred to as a base-to-apex sequence of excitation spread [35]. Once ventricular septation is complete, the sequence of activation switches from base-to-apex to an apex-to-base pattern. This shift in signal propagation typically marks the start of the mature ‘apex-first’ excitation [35].

### 2.9. Congenital Heart Malformations

Cardiac malformations ensue when developmental processes become abnormal. Developmental mechanisms leading to anomalies in mesenchymal tissue migration, cardiac hemodynamics, and ECM deposition are responsible for most cardiac malformations [36]. The specific defects that form depend on the disrupted developmental process. For example, a perimembranous ventricular septal defect (VSD), the most common congenital heart defect in humans, is thought to be the result of an imbalance of intracardiac blood flow that affects completion of the ventricular septation process. Similarly, flow imbalance is responsible for other CHDs, such as coarctation of the aorta (CoA), pulmonary atresia with intact ventricular septum, and aortic valve stenosis.

Abnormal endocardial cushion development is presumably the basis for CHDs known as endocardial cushion defects or atrioventricular septal defects. When the anterior and the posterior AVC endocardial cushions do not fuse, division of the AVC into the tricuspid and mitral orifices fail, resulting in defects in the atrioventricular valves and septum that are commonly seen in children with trisomy 21 or Down syndrome. In contrast, a limited fusion failure of the OFT conal cushions is responsible for subarterial VSDs.

A broad spectrum of CHDs, known as conotruncal malformations, are caused by abnormal development of the heart OFT and account for about 30% of CHDs in humans. In general, conotruncal malformations occur due to abnormal mesenchymal tissue and/or neural crest cell migration. This group of CHDs include conditions such as aorticopulmonary window (APW), in which abnormal septation of the conotruncus above the aortic and pulmonary valves results in an open communication between the aorta and pulmonary artery. In tetralogy of Fallot (TOF), a CHD consisting of VSD, right ventricular hypertrophy, pulmonary valve stenosis, and over-riding aorta, a right shift in the APS is believed to be the culprit process. Furthermore, the most common cardiac cause of neonatal cyanosis, d-transposition of the great arteries (d-TGA), where the aorta is aligned with the right ventricle and the pulmonary artery is aligned with the left ventricle (transposed) occurs as the OFT cushions are positioned in a straight line instead of in a spiral path. Another typical CHD caused by abnormal tissue migration is double outlet right ventricle (DORV), where both aorta and pulmonary arteries completely or almost completely emerge from the right ventricle. Anatomic variations in DORV, such as the location of the septal defect relative to the great vessels, generate a wide range of clinical manifestations, from a simple shunting physiology, mimicking a VSD, to a cyanotic disease, such as TOF or d-TGA, with significant surgical treatment implications. The extreme CHD member of the conotruncal malformation spectrum, truncus arteriosus, is caused by absence of aorticopulmonary septation, and is clinically like APW in manifestation.

Any developmental disruption at any stage has hemodynamic consequences that affects the pathophysiology of CHD. The continuous interaction between morphogenesis and blood flow is responsible for the final cardiac anatomy: morphological changes result in hemodynamic change, which leads to remodeling and further morphological changes, either as a successful or unsuccessful compensatory process. For instance, in hypoplastic left heart syndrome (HLHS), the low to no flow in the underdeveloped left ventricle is compensated by an overdeveloped right ventricle. Similarly, the high resistance to flow in coarctation of the aorta (CoA) diverts the flow to the ductus arteriosus, maintaining it widely patent and large (frequently the size of the ascending aorta) in the newborn. Therefore, hemodynamic effects in CHD cannot be neglected.

## 3. Animal Models of Heart Development

To study the role of blood flow (hemodynamics) on heart development, different animal models have been used, such as zebrafish, chicken, mouse, and sheep [37]. Because genetic processes are highly conserved among vertebrates, studying these animal models has greatly contributed to the understanding of heart development. Studies seeking to determine how hemodynamics affects development should separate blood flow effects from other effects (e.g., genetic predisposition or drug responses). This could be difficult to accomplish, and most studies discuss results that are confounded by several factors. Nevertheless, the aggregate combination of studies and animal models has begun to reveal the complex interactions between blood flow and genetic programs.

Mammalian models of heart development (e.g., mouse and sheep), closely reproduce conditions during human development. Like in humans, a placenta in mammals allows for exchange of nutrients and oxygen from the mother to the developing baby. However, experimental access to the embryos and fetuses inside the womb is challenging. In ovine models the fetus is relatively large at late stages of development, and by using challenging invasive procedures, the fetal lamb heart can be both intervened and instrumented. For example, studies in sheep found that an increased hemodynamic load during fetal stages (e.g., by ligating the aorta) results in an enlarged heart [38,39,40], while reduced left ventricular preload (by occluding the foramen ovale) leads to left ventricular hypoplasia [41,42]. Nevertheless, working with ovine models can be costly and even though the developing fetus is relatively large, experimental access inside the womb is difficult.

Among the developmental research community, mice are a preferred model to study heart formation with the possibility of performing genetic manipulation and cell tracing during development [43]. By taking advantage of mutant lines, studies in mice are elucidating the effects of individual genes on embryonic and cardiac development. Among those, genes involved in sensing and responding to blood flow, as well as genes that regulate cardiac contraction and function, have been studied [44,45]. However, minimally invasive access to mouse embryos for in vivo imaging and follow up studies remain extremely challenging and not practical. Thus, mouse mutants and inducible lines have been mainly used to characterize the role of specific genes in cardiac dysmorphogenesis [46].

To mitigate limitations in accessing the embryo, cultured mouse embryos have been used [47,48]. However, the technique is limited to early stages of development. Recent advances in ultrasound bio-microscopy may allow for studying fetal mouse hearts in vivo and delineate the relationship between cardiac morphology and function in mouse mutants. In addition, current genetic technologies allow targeted deletion or overexpression of genes in specific cell populations, as well as the generation of inducible lines that can temporally silence or overexpress genes at specific windows of time. Together, these technologies are enabling determination of how genes affect heart morphology and function over developmental stages [46], opening exciting new opportunities for in vivo follow up studies in mice.

Zebrafish is another popular model of heart development [49] given that genetically manipulated lines are available, and hundreds of embryos can be obtained from fertilized eggs. Moreover, the early embryos are semitransparent and thus blood flow and cardiac motion from transgenic lines with fluorescent red blood cells, and/or fluorescent endocardial or myocardial cells, can be easily imaged in vivo [50]. Using transgenic and mutant lines, the effects of an array of genes on heart development have been studied. These include genes affecting cardiac contractility as well as genes that sense and respond to blood flow [51,52]. Examples of studies performed in zebrafish to unravel interactions between blood flow and gene networks include changes in hematocrit to alter blood viscosity and wall shear stress, drug treatment or genetic manipulations to impair cardiac contraction affecting blood flow dynamics, centrifugation of embryos affecting blood flow, and occlusion of the heart outflow and inflow with glass beads to reduce or interrupt blood flow to the heart [8,49,50,52,53]. While much has been learned from the teleost model of heart development, an important limitation of zebrafish models is that the adult heart has only two chambers, and thus findings are mostly applicable to early stages of human cardiac development.

The chicken model of heart development has several advantages over other models. The embryos start developing once eggs are placed in an incubator, allowing control of timing. Chicken embryos are easy to image in vivo, allowing measurement of blood flow velocities and cardiac motion. Unlike zebrafish, genetic manipulations in avian models are difficult, but the chicken heart develops into a four-chamber heart with valves allowing for replication of several heart defects found in humans. Different techniques in chickens, including quail-chick chimeras, have made possible cell tracing studies to elucidate the fate of extra-cardiac cell contributions to heart development (e.g., the contribution of neural crest cells to the formation of the heart). Moreover, it is relatively easy to manipulate chicken embryos either via administration of drugs or other bioagents, or by surgically altering blood flow conditions. Chicken embryos have been widely used to alter blood flow in otherwise normal embryos using surgical interventions [9,10]. The chicken embryo model allows for studying blood-flow induced changes that lead to heart defects frequently found in humans. In this review, we mainly focus on findings from chicken models of heart development, as they replicate human heart defects in response to altered blood flow conditions.

## 4. Blood Flow during Cardiac Development

Blood flow plays a critical role in heart formation [8]. The developing heart is sensitive to changes in flow, while distinctly responding to different levels of hemodynamic load [10,54]. Although genetic components have proved to be one of the CHD causal factors, only 20% of heart defects are linked to genetic mutation [55,56]. Cardiac defects are considered a compound consequence of both genetic and environmental factors. Blood flow and heart structure reciprocally influence each other. The developing embryonic heart remodels in response to mechanical cues from blood flow (e.g., biophysical forces that generate tissue stresses), and the resultant cardiac structural modifications affect and alter back blood flow.

### 4.1. Hemodynamic Stresses and Mechanotransduction

**Figure 3:**
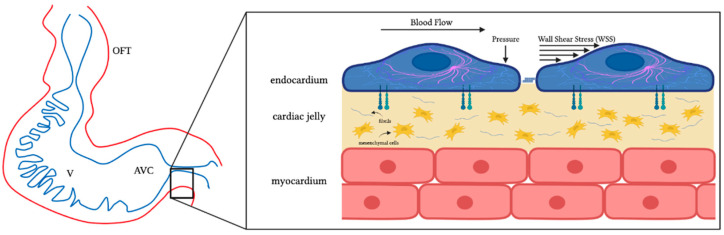
Schematics of the tubular developing heart and cardiac walls. The figure shows a sketch of the looping tubular heart (**left**) as well as a sketch of the microstructure of its wall (**right**). Endothelial cells line the interior of the heart wall, forming an endocardium layer. Cells in the endocardium are in contact with blood and directly exposed to blood flow stresses, wall shear stress and blood pressure. Stresses and deformations are sensed through ion channels, the cell cytoskeleton, cell–cell junctions, and cell adhesion proteins among other mechanisms. Myocardial muscle cells line the exterior of the cardiac wall forming a myocardium layer. In between the endocardium and myocardium, there is a cardiac jelly layer. In the cushions, the cardiac jelly layer has mesenchymal cells. While the myocardium is not in direct contact with blood, it senses stresses and is deformed due to its contraction–relaxation behavior and its interaction with blood flow. OFT: outflow tract; V: ventricle; AVC: atrioventricular canal. Created with BioRender.com (accessed on 24 July 2022).

Blood flow is established and sustained shortly after the formation of heart tube [8]. Biophysical forces resulting from the interaction of tissue with blood flow generate tissue stresses that affect cardiac development and maturation. Stresses exerted by blood flow on cardiac tissues include (1) blood pressure, which is perpendicular to the cardiac tissue surface, and (2) wall shear stress (WSS), which arises due to the friction of flowing blood on the endocardial lining surface (Figure 3) [57]. Together, these stresses characterize the cardiac ‘hemodynamic load’ and play a critical role in cardiac cells’ functions. As described in Section 2, the early heart tube consists of three layers (endocardium, myocardium, and cardiac jelly) which respond uniquely to hemodynamic loads.

**Figure 4:**
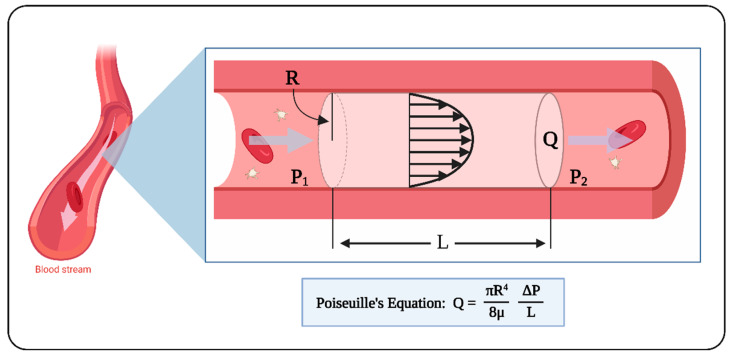
Laminar flow of an incompressible fluid inside an axisymmetric vessel. Under steady state conditions, the volumetric flow rate, Q, is described by the Poiseuille equation (Equation (1)). The velocity profile is parabolic, and the fluid flows from higher to lower pressure. Poiseuille flow can be used to approximate blood flow. ΔP = P_1_ − P_2_; L: length; R: radius; μ: fluid viscosity. Created with BioRender.com (accessed on 24 July 2022).

To better understand the relationship between blood flow and cardiac tissue stress-es, let us consider simple, yet insightful, cases. The motion of fluid within a tube is a classic example of flow conditions applicable to cardiovascular blood flow. Laminar, steady flow within an axisymmetric (cylindrical) vessel (Poiseuille flow; see Figure 4) is characterized by a parabolic flow velocity profile, and constant pressure over cross-sectional planes that decrease linearly along the vessel (pressure gradient). The relationship among the flow rate (Q), change in pressure (ΔP) along the vessel length (L), and WSS is described as:

(1) $$Q\;=\frac{{\pi \;R}^{4}}{8\;\mu }\;\frac{\Delta P}{L}$$

(2) $$\mathrm{WSS}=\;\frac{4\;\mu }{{\pi \;R}^{3}}\;Q\;$$

where R is the axisymmetric vessel radius, and μ is the flow viscosity. When we use these equations to approximate conditions in a blood vessel or even in the tubular heart, under a constant pressure gradient, the flow rate (Q) within the vessel is proportional to R^4^ (Equation (1)), and thus Q will vary substantially with any small change in the vessel radius. Alternatively, if Q remains constant, then both WSS and ΔP vary considerably when R changes. These are important considerations when dealing with changes in blood vessel dimensions, either due to malformations or as a way to alter hemodynamic conditions.

**Figure 5:**
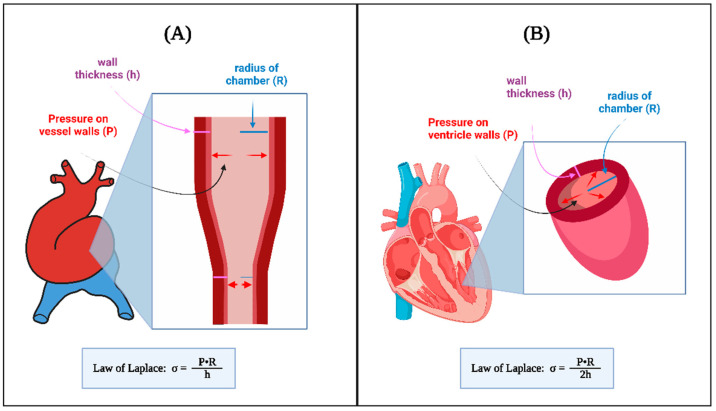
The law of Laplace relates transmural pressure P to circumferential wall stress σ. In a developing tubular heart (**A**) and a spherical model of a ventricle (**B**) there is a proportional relationship between circumferential stress, σ, and the fluid pressure acting on the walls, as well as the radius R. There is also an inversely proportional relationship between circumferential wall stress and wall thickness, h. While approximate, Laplace law can be used to estimate wall stress values, and the effects of P and heart dimensions on wall stress. Created with BioRender.com (accessed on 24 July 2022).

Fluid pressure (P) within a circular cylinder generates circumferential stresses (σ) on the walls of the cylinder or blood vessel (see Figure 5A). Circumferential stresses resist the deformation of the vessel (changes in tube radius R). Under equilibrium conditions, and assuming the vessel wall is thin and σ is constant along the wall thickness, h, relationships between circumferential stress and pressure follows the Laplace law:

(3) $$\sigma \;=\frac{P\cdot R\;}{h}\;$$

Similarly, if instead of an axisymmetric vessel we consider a sphere of radius R filled with a fluid at the pressure P, a rough approximation of stresses in the heart ventricle (see Figure 5B) the Laplace law becomes:

(4) $$\sigma \;=\frac{P\cdot R\;}{2h}\;$$

Thus, altering either P or R changes the circumferential stresses, σ, to which tissue cells are exposed (Equations (3) and (4)). Together, Equations (1)–(4) can be used to approximate the consequences of altered heart/blood vessel dimensions on blood flow.

Endocardial cells are in direct contact with blood and sense hemodynamic stresses via membrane-embedded molecules, such as G-protein coupled receptors, ion channels, and primary cilia that are localized at the cell surface [58,59,60]. Upon contact with blood, these membrane-bound molecules are physically deformed and convert this mechanical change into a biological signal that activates genes, such as KLF2, ET-1, and NOS-3, which are particularly sensitive to WSS and are essential for the activation of downstream mechanotransduction pathways. Additionally, many membrane-bound and cell-cell adhesion molecules are interconnected with the cytoskeleton. When experiencing WSS, these molecules produce tension on the cytoskeleton and signal the cell to activate downstream signaling pathways. Endocardial cells are also able to signal and modify the development and function of the underlying myocardium. For example, ET-1 secreted by endocardial cells acts on the myocardium to increase contractile force and heart rate [61,62].

While not in direct contact with blood flow, myocardial cells, or cardiomyocytes, also experience cyclic wall stress (as well as stretch) with each contraction-relaxation cycle, affecting cardiac development [57]. Like endocardial cells, cardiomyocytes have membrane-bound molecules that respond to mechanical stimuli [63]. Moreover, myocardial cells can alter their shape and orientation in response to stress, such that cardiomyocytes tend to orient and elongate in the direction of stretch [64], augmenting contraction efficiency. There is still much to learn about the way cells in the developing heart sense and respond to varying levels of stress.

### 4.2. Hemodynamic Interventions in Avian Models

**Figure 6:**
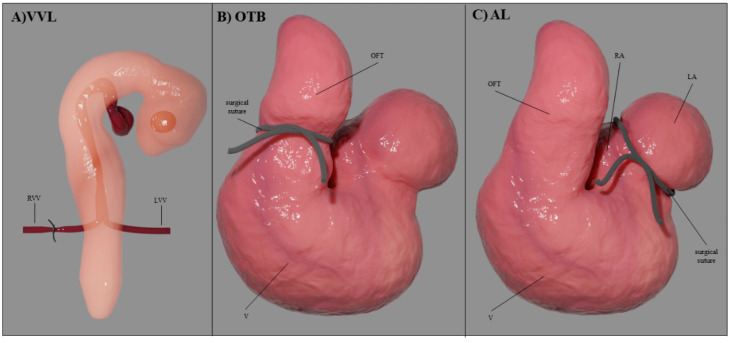
Hemodynamic interventions performed on chick embryos. (**A**) A surgical suture (or clip) is placed and tightened around one of the two vitelline veins that feed the heart at HH17-18. (**B**) A surgical suture is placed and tightened around the heart OFT at HH17-24. (**C**) A surgical suture (or clip) is placed and tightened around the left or right portion of the developing atria at HH21-24. VVL: vitelline vein ligation; OTB: outflow tract banding; AL: atrial ligation: RVV: right vitelline vein; LVV: left vitelline vein; OFT: outflow tract; V: ventricle, LA: left atria, RA: right atria.

Several experimental interventions have been introduced in chicken embryos to perturb the normal blood flow conditions by means of surgical procedures. For a clear reference to developmental stages, avian development is described according to the Hamburger-Hamilton (HH) staging system [65]. The HH stages are associated with specific embryonic processes and morphological characteristics, allowing for accurate reference to all developmental events. The tubular heart starts beating around HH10 (≈35 h of incubation) and cardiac septation and valve formation begin after HH24 (≈day 4) once the heart looping is complete. Endo-MT starts around HH15 in AVC cushions and around HH17 in OFT cushions with neural crest cells entering the OFT cushions around HH21 (see Figure 2). By HH34 (≈day 8), the heart is fully septated with valves already formed. Surgical interventions are typically performed during tubular stages of heart development (see Figure 6) and include: vitelline vein ligation (VVL) or vitelline vein clipping; outflow tract banding (OTB), which is sometimes referred to as conotruncal banding; and atrial clipping or ligation (AL) [9]. These surgical interventions disrupt blood flow (see Table 1) allowing us to study how abnormal hemodynamics affect the development of the heart.

In VVL, one of the two vitelline veins (right or left) that feed blood to the primitive tubular heart is ligated or clipped [66,67]. The intervention is performed early during tubular heart stages, typically at HH17 or HH18. The ligation or clipping stops blood flow from the vein, leading to an acute reduction in blood flow (Q) to the tubular heart, thus reducing WSS and pressure in the heart (Equations (1) and (2)) as well as establishing a right-to-left flow imbalance. The effects of VVL last up to 5 h, before angiogenesis re-routes blood flow to the heart. VVL results in a broad spectrum of heart defects, especially conotruncal abnormalities, including VSD and DORV. It can also lead to malformations of the semilunar valves and pharyngeal arch arteries (PAAs) [67].

In OTB, the OFT is ‘banded’ by means of a surgical suture placed and tightened around the OFT that effectively reduces the lumen area by constricting the OFT diameter [68,69]. OTB is performed during tubular stages of heart development, typically between HH17 and HH24, with the suture removed later to increase the embryo’s survival. While OFT diameter is reduced by the band, stroke volume remains constant, such that Q remains approximately constant. Due to the constriction, OTB increases the resistance to blood flow rising ventricular blood pressure (Equation (1)) and elevating systemic blood pressure, while increasing WSS in the region where the surgical suture (band) is located (Equation (2)). OTB leads to abnormal cushion development and conotruncal heart defects, including VSD, DORV and TOF [10,68].

In AL, either the right (RAL) or the left (LAL) portions of the developing atria are ligated or clipped [70]. The intervention is typically performed early during tubular stages of development at HH21 or HH24. The ligation or clipping excludes a portion of the atria from the circulation, reducing blood flow and producing a right-to-left flow imbalance. Because of flow redirection to the right side after LAL, right atrioventricular valve morphology can be altered from a normal muscular flap to a bicuspid structure. Moreover, due to the reduced Q and WSS, LAL embryos develop HLHS at HH34, characterized by an underdeveloped left ventricle [71,72]. Conversely, RAL embryos show left ventricular hyperplasia (increased myocyte proliferation) and relatively small right ventricles [72].

Hemodynamic perturbations in chicken embryos lead to changes in the heart morphology, size, mass, cushion formation, ventricular trabeculation and trabecular architecture, as well as aberrant septation and valve development. We will discuss some of these adaptations in more detail below.

### 4.3. Computational Modeling and Analysis of Heart Hemodynamics

Physics-based computational modeling is an insightful tool to study blood flow during cardiac development events [50,73]. Computational models are used to solve the governing equations of flow within the heart and quantify blood flow velocities and flow stresses. When explicitly modeling the interaction between the cardiac walls and the blood flow, fluid-structure interaction (FSI) strategies are used. FSI models the movement of the contracting cardiac walls and the blood flow within the heart and can be a precise method if the mechanical properties of the cardiac wall are known. However, the mechanical properties of the developing heart are typically hard to estimate. Cardiac walls have both active (e.g., active contraction) and passive (e.g., passive relaxation) properties that are difficult to measure, as they are not isotropic, and significantly change over developmental stages and under pathophysiological conditions. These difficulties make assessing wall properties challenging.

Alternatively, computational fluid dynamics (CFD), which solves for the blood flow within the lumen of the heart, can be used. To account for the motion of the cardiac walls during cardiac contraction and relaxation, the boundaries (contour surfaces) of the CFD model are displaced in a prescribed fashion that recapitulates cardiac wall motion. In this way, by introducing a dynamic CFD geometry, idealized or image-based cardiac wall motions are incorporated into CFD models of the beating heart. CFD is generally the method of choice as it is computationally less expensive than FSI and does not require knowledge of the mechanical properties of cardiac walls.

When modeling the dynamics of blood flow, rheological characteristics of blood together with the dimensions of the developing heart need to be considered in choosing the right computational and mathematical model of blood flow. Blood consists of plasma and cells. While plasma is a Newtonian fluid with a constant dynamic viscosity, the presence of red blood cells (RBCs) makes blood a non-Newtonian fluid, with a dynamic viscosity that depends on both the hematocrit, or percentage of RBCs in blood, and the shear rate of the flow [74]. For vessels with diameters comparable to those of RBCs (e.g., capillaries), the motion and deformation of individual RBCs must be considered. During embryonic development, the characteristic dimensions of the heart, flow rates, shear rates, and hematocrit constantly change, making it challenging to choose the right flow model. When modeling blood flow, Newtonian models are typically used both during early development, when hematocrit is low, and during late developmental stages, when the heart is fully formed, and shear rates are significant. However, blood viscosity is challenging to estimate, and validation of modeling results against experimental measurements of blood flow velocities are necessary. Nevertheless, CFD is a useful tool for quantifying the detailed spatial and temporal distribution of blood flow pressure gradients and velocities, from which WSS (which cannot be measured) is computed [75].

#### 4.3.1. Modeling Hemodynamics during Cardiac Cushion Formation

Several CFD studies have investigated blood flow parameters in the tubular heart. One of the first such studies considered an idealized computational model of the heart tube to determine the effect of endocardial cushions on cardiac function [76]. The presence of two opposing endocardial cushions on the heart tube wall early on during development induces a transition to pulsatile flow before valves are formed. The study highlighted that cushion morphology facilitates lumen closure to establish unidirectional flow in the primitive tubular heart.

Other studies have used image-based CFD models with moving walls. Among those, 2D CFD models of the zebrafish AVC over developmental stages revealed that blood flow through the AVC, and the associated WSS, are initially bi-directional [77]. As the heart continues to develop, flow gradually becomes unidirectional, while WSS and the magnitude of the pressure gradient through the AVC increase. A 3D CFD study based on dynamic 3D images and moving walls (also referred to as 4D models) of the chicken embryo OFT showed that, as the endocardial cushions grow over developmental stages (HH14-HH18), blood flow through the OFT becomes unidirectional [78]. The study further revealed that HH16 is a period of OFT hemodynamic transition in chicken embryos. As the OFT cushions become more efficient at preventing backflow, regions of higher WSS transition from the OFT’s distal to proximal regions. This hemodynamic transition coincides with initiation of Endo-MT in OFT endocardial cushions along with an increase in the OFT diameter [22,27]. Changes in WSS accompanied by increased diameter might reflect early mechanobiological mechanisms that limit WSS levels in the developing heart [22].

Dynamic 4D image-based models of the OFT were also used to determine the WSS distribution at HH18, right after OTB hemodynamic intervention [79]. Results revealed that WSS is heterogeneously distributed through the endocardial wall surface with patterns like those of control embryos, but the elevated WSS in OTB is due to the increase in blood flow velocity, particularly on the cushion region. This increased WSS on cushions could lead to abnormal Endo-MT and the conotruncal heart defects observed post OTB [10].

#### 4.3.2. Modeling the Septation Stages

CFD models of septation have been used to capture the synergistic changes in blood flow patterns due to the growing septa and to model the influence of blood flow on septation. In chicken embryos, ventricular and OFT septation start after HH24. A study used a 4D ultrasound acquisition strategy to generate image-based moving-wall CFD models of the HH25 chicken heart [80,81]. Analysis of the 4D CFD model simulations highlighted the differences in WSS between the two sides of the heart, with the left portion of the heart (the future left ventricle) exhibiting lower WSS than the right portion (destined to be the right ventricle) [80]. WSS was elevated in the AVC, the OFT, as well as near the ventricular and atrial septa where the lumen narrows [81]. Interestingly, at HH25, ventricular WSS in the chicken embryo was found to be the same order of magnitude as WSS of human fetus at 20 weeks of gestation [82]. While at HH25 the avian heart begins to septate, at 20 weeks of gestation the human heart is fully formed and septation is complete. The similar WSS values might reflect mechanobiological mechanisms that limit WSS levels in the developing heart. Future studies shall determine whether WSS levels in the developing heart trigger growing mechanisms that limit WSS.

Other CFD studies suggest that at HH27, a change in the characteristics of the flow precedes the formation of the APS in the OFT [83]. The previously aligned flow through the OFT adopts a spiral-dominated pattern due to the mixing of the input volumes coming from the recently separated ventricles. This spiral flow characteristic gets attenuated at HH30 in regions near the developing APS wall. An increase in the WSS of the OFT is observed at stage HH30 that coincides with APS formation and is directly proportional to its growth [83]. Thus, OFT septation leads to changes in hemodynamic patterns that can drive proper division of the OFT into pulmonary and aortic arteries and their valves.

To characterize changes in flow dynamics leading to HLHS, LAL was performed at HH21, and CFD models corresponding to stages HH21 and HH30 were generated [70]. CFD models used microCT images of fixed hearts to extract the AV lumen geometry and its surroundings. This strategy led to static image-based CFD models, with simulations that were accurate mostly during peak flow conditions. To simulate changes in blood flow, Doppler ultrasound measurements performed on embryos were imposed in CFD models as flow velocity boundary conditions, and non-Newtonian blood flow properties were used [75]. At HH21, right after LAL, CFD simulations revealed an abrupt decrease in WSS (with respect to pre-LAL conditions) on the AVC surface, especially on its left side, concurrent with a decrease in flow from the primitive atrium to the primitive ventricle due to LAL. At HH30, when left and right atrioventricular connections have formed, CFD simulations revealed that LAL leads to a WSS imbalance between the left and right AVCs, with lower WSS levels in the left AVC and higher WSS in the right AVC. This contrasts with control hearts in which WSS levels are similar in the right and left AVC at HH30. The redistribution of flow towards the right side of the heart due to LAL correlates with the underdevelopment of the left ventricle and overdevelopment of the right ventricle, demonstrating the significance of disturbed hemodynamics during ventricular development.

CFD has proven to be a useful approach to understand how hemodynamics affect cardiogenesis. When used together with experimental measurements, CFD provides a link among flow, tissue growth, and tissue remodeling during development that can be further used to explore mechanobiological mechanisms that regulate heart development.

## 5. Effect of Hemodynamics on Cardiac Development and Formation

A spectrum of heart defects originates from abnormal blood flow during early developmental stages (see Section 4.2). In what follows we will discuss details of the known effects of abnormal blood flow on heart formation.

### 5.1. Formation of Endocardial Cushions

Endocardial cushions are the precursors of heart valves and septa. When cushion development and maturation go awry, heart septation and valve formation are compromised. While molecular mechanisms directing cushion formation have been extensively studied, less is known about how hemodynamic stresses affect signaling processes and cushion development. Endocardial cushions bulge into the OFT and AVC of the developing heart (see Figure 3), exposing them to a local increase in blood flow velocity, Q, and WSS. Subsequent cushion cellularization through Endo-MT is affected by WSS.

**Figure 7:**
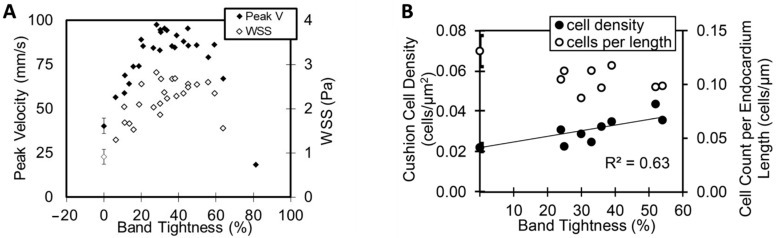
Outflow tract banding (OTB) intervention increases endocardial cushion mesenchymal cell counts in the heart OFT cushions. OTB was performed on chicken embryo hearts at HH18, and the OFT imaged in vivo before and after intervention; embryos were then collected at HH24 (after 24 h of OTB) and their hearts imaged. (**A**) Peak (maximum) blood flow velocity measured in vivo in the OFT 2 h after OTB, and estimated WSS on cushions, as a function of surgical suture (band) tightness, measured as a percent reduction in maximum OFT diameter due to the band. (**B**) Quantification of OFT endocardial cushion cell density and number of cells per unit endocardium length as a function of band tightness at HH24 after 24 h of OTB. A 0% band tightness corresponds to the control group. Adapted and reproduced from [27,84], respectively, under Creative Common License.

An increase in WSS in OFT cushions correlates with increased cushion cell density in chicken embryos. In OTB, the tightness of the suture around the OFT (band tightness) dictates the increase in WSS on OFT cushions (Figure 7A and Equation (2)) and determines the increase in cell density in OFT cushions (Figure 7B) [84]. Whether the increased cell population in OTB is due to an increase in the number or rate of endocardial cells undergoing Endo-MT or due to enhanced proliferation of cells in the cushions is not yet clear. Studies suggest that elevated WSS leads to increased number of successfully transitioning cells [27,52].

Hemodynamic loading continues to contribute to cushion maturation even after Endo-MT is complete. In chicken embryos, after banding (OTB), the increased WSS on endocardial cushions is associated with disorganization of ECM proteins typically found within the endocardial cushions, e.g., tenascin, fibrillin, and collagen [85,86]. Additionally, increased WSS reduces the levels of apoptosis within the cushions, which is crucial for AVC and OFT tissue remodeling [85]. Because endocardial cushions are the precursors of valves and septa, abnormal blood flow not only affects cushion formation but can later lead to valve and septal defects.

### 5.2. Ventricular Formation

**Figure 8:**
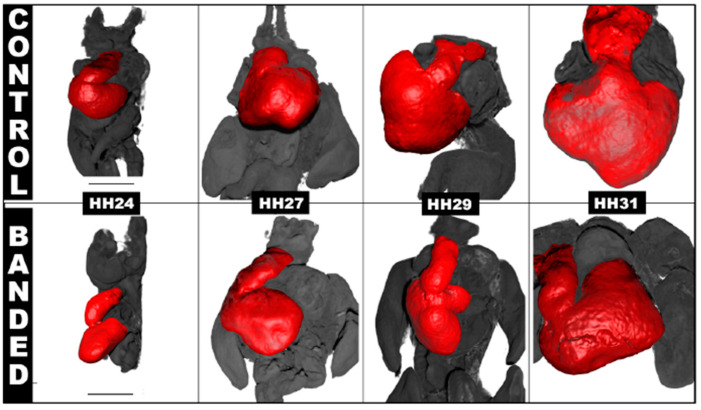
Reconstructed anatomies of chicken embryo hearts at different developmental stages from 3D microCT scans. The figure compares normal heart development (control) with abnormal cardiac development due to OTB (banding intervention). Morphologically, the banded hearts tend to have a bean-like shape form when compared to the control hearts. This is perhaps more visible in the HH24 and HH29 images here, in which the control hearts are approximately 1.5-fold wider than banded hearts with surface features that portray the ongoing septation of the primitive ventricle. Scale bar is 1 mm, and all images are the same scale.

Ventricular formation highly depends on hemodynamic stresses within the developing heart, and abnormal blood flow can lead to ventricular adaptations that impair cardiac function. In OTB embryos, elevated hemodynamic load leads to an increase in myocardial proliferation (compared to controls) and accelerated rate of ventricular growth [87]. This initial accelerated rate of growth is followed by a spectrum of changes in ventricular and heart morphology over developmental stages (e.g., Figure 8). Some studies reported that the ventricles adapt to a more ‘ideal’ spherical shape, while others reported no changes to the overall morphogenesis of the ventricle [88,89].

Atrial ligation (AL) generates an imbalance of flow between the right and left sides of the heart, early during development that alters ventricular proportions. In LAL performed at HH21, a redistribution of flow to the right side of the heart leads to underdevelopment of the left side (hypoplasia of the left ventricle) with compensatory overdevelopment of the right side as soon as HH29. Left ventricle myocardial volume decreases while left side trabecular compaction accelerates. The right ventricle shows chamber dilation with altered trabecular pattern and proliferation [71]. In contrast, in RAL, the flow imbalance leads to overdevelopment of the left heart and underdevelopment of the right heart.

**Figure 9:**
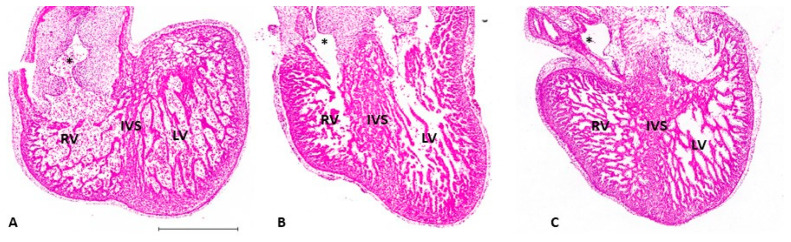
Histological images of the developing ventricles at HH29 demonstrate different ventricular morphology following OTB. Sectioned chicken embryonic hearts at HH29 were stained with Hematoxylin (nuclear marker) and Eosin (cytoplasmic maker) and imaged at 20× magnification. The figure compares the ventricles of (**A**) a control heart, (**B**) an OTB heart with a band tightness of 42%, and (**C**) an OTB with a band tightness of 47%. Hearts subjected to OTB (**B**,**C**) exhibit denser trabeculae and increased compact myocardium thickness. The heart subjected to 42% band tightness (**B**) demonstrates a narrowing of the ventricle towards the apex of the heart, while the heart subjected to 47% band tightness (**C**) demonstrates a more spherical shape. LV: left ventricle; RV: right ventricle; IVS: interventricular septum; *: start of OFT. Scale bar = 500 µm.

Hemodynamic load also affects trabecular architecture. In OTB, elevated hemodynamic load results in an acceleration in trabecular compaction when compared to control embryos at the same stage. The increased myocardial proliferation and trabecular compaction leads to a thicker ventricular wall, together with a denser and more spiraled trabecular pattern [71,88]. Varying band tightness, which leads to varying levels of hemodynamic load, contributes to a spectrum of changes observed after OTB both in shape and trabecular architecture (e.g., Figure 9).

Embryos that experience a decrease in hemodynamic load, such as those intervened through VVL and LAL, exhibit opposite effects compared to OTB embryos. Following VVL, ventricular growth is delayed, and the compact myocardium is thinner with reduced ventricular trabeculation compared to normal hearts [90]. Similarly, embryos that underwent LAL show delays in ventricular growth, with ventricles being significantly smaller compared to controls [71].

**Figure 10:**
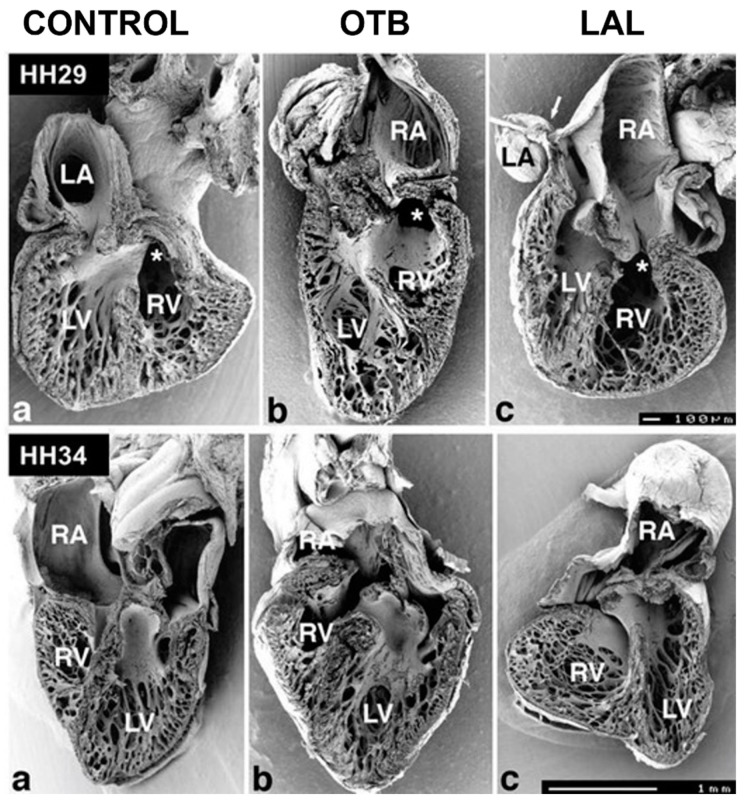
Scanning electron microscopy images of developing chicken hearts. The pictures show halves of frontally dissected hearts at HH29 (**top, ventral halves**) and HH34 (**bottom, dorsal halves**). For both stages, control and hemodynamically intervened hearts are shown. (**a**) Control hearts; (**b**) conotruncal banded hearts (OTB); (**c**) left atrial ligation (LAL) hearts. **Top (HH29):** Compared to the control heart, the OTB heart is more elongated with spiraling trabeculae, while the LAL heart exhibits closer packed trabeculae. Stars marks the entry of the conotruncus. Scale bar = 100 µm. **Bottom (HH34):** Compared to the control heart, the atria of the OTB and LAL hearts are distorted, and ventricular proportions changed. Trabecular structures also show a distinct architecture. Scale bar 1 mm. RA: right atrium; LA: left atrium; RV: right ventricle; LV: left ventricle. Reproduced with permission from [71].

**Figure 11:**
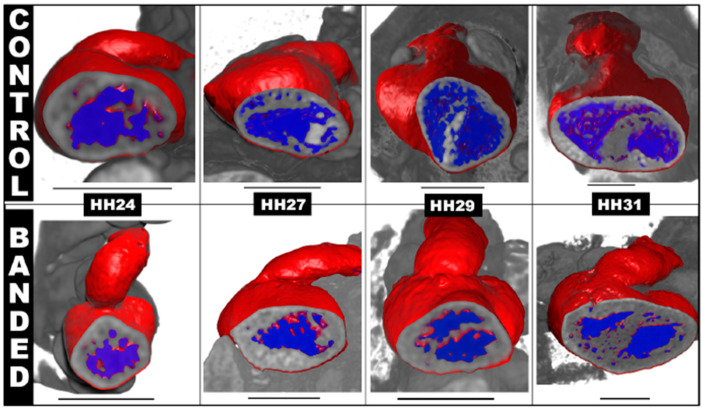
Ventricular growth and septation during embryonic heart development via 3D reconstruction of microCT scan data. The images focus on the septation of left and right ventricles in the embryonic chicken heart at different stages of development. At HH24 (after 24 h of OTB or banding intervention), the suture is removed. The primitive ventricle is not yet septated. At later stages, the banded hearts form a larger left ventricle and smaller right ventricle, compared to control hearts. At HH31, the banded heart here shows a clear septum between right and left ventricles accompanied by increased trabeculation. Scale bar is 1 mm for all images.

Overall, ventricular formation highly depends on blood flow characteristics. An increase in hemodynamic load results in accelerated rates of ventricular growth, while a reduction in hemodynamic load leads to the opposite (see Figure 10 and Figure 11). Further investigation is needed on the effect of hemodynamics on ventricular signaling pathways and how hemodynamics around trabeculae dictates their architecture.

In clinical practice, increased load through arterial banding is sometimes used for ventricular ‘training’. In patients with corrected transposition of the great vessels, the ventricular switch operation aligns the weaker right ventricle to the aorta, facing systemic pressures, which will subject it to failure. Instead, a controlled higher afterload imposed on the ventricle by the band induces myocardial hypertrophy and will prepare it to work as a systemic ventricle.

Morphological changes from the interaction of heart tissues with blood flow or due to loading are well-known and used in surgical treatment of CHDs. For instance, in some newborns with TOF-where the peripheral pulmonary arteries are more hypoplastic than expected-the complete surgical repair by VSD closure and relief of the stenosis at the right ventricle outflow tract and pulmonary valve is avoided due to the concern of having the right ventricle facing high downstream resistance from the underdeveloped peripheral pulmonary arteries. In these patients, a palliative surgery is conducted with implantation of a systemic-pulmonary shunt, such as Blalock Taussig shunt, subjecting the small pulmonary vasculature to systemic pressures and flows that promote accelerated growth until the second surgery for complete repair.

Abnormal hemodynamic load can lead to septal defects. In VSD, the interventricular septum is incomplete, precluding separation of blood in the left and right ventricles as occurs in a normal heart. The incidence of VSD after OTB can be between 76% and 100% and depends on the duration of banding period [68,71] and band tightness [10]. In VVL, VSD incidence can be as high as 70% [67]. In LAL, VSD is found in about 25% of hearts, but with a mild representation [71]. Thus, it appears that ventricular septation is highly sensitive to blood flow conditions, with both higher and lower than normal hemodynamic loads leading to VSD.

### 5.3. Outflow Tract and Great Vessels

**Figure 12:**
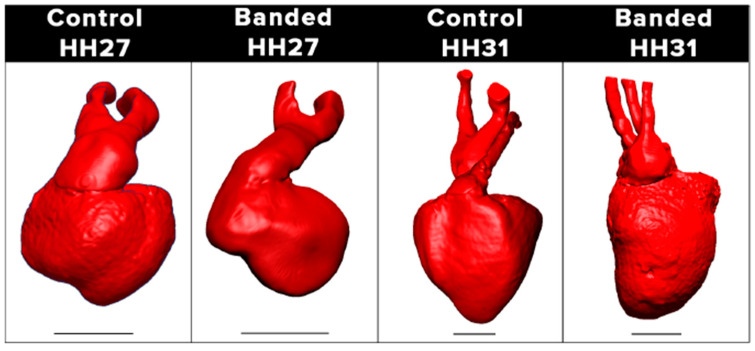
Reconstructed anatomies of chicken embryo hearts and the great arteries at HH27 and HH31 from 3D microCT scans. The images compare the septation of the OFT, with both stages showing separation of pulmonary and aortic outflows. Abnormal outflow positioning is observed in banded hearts at HH27 and HH31. Banded HH27 heart was captured at a side angle to better view the OFT, which exits the heart at a different angle than the control heart. Differences in the position of great arteries are more prevalent at HH31. Scale bars are 1 mm.

The specific role of hemodynamics in OFT septation remains unclear. Several studies have suggested a correlation between a change in flow behavior and APS formation [83]. Both VVL and OTB [9] lead to disturbed cushion formation and eventually a heart with malpositioned arterial and pulmonary trunks (e.g., Figure 12). As mentioned before, proper rotation and alignment of the OFT, and its septation, depend on the contribution of extra-cardiac cells. Whether migration and proliferation of precursor cells is impaired by abnormal blood flow, leading to malposition of the great arteries and septation anomalies, is not yet known.

### 5.4. Development of the Conduction System

In normal chicken embryos, the mature apex-first activation pattern is observed at HH36, although this activation pattern can be seen as early as HH29 in a small portion of the hearts [91]. To test the effect of hemodynamic load, OTB was performed at HH21 to increase hemodynamic load, while LAL was performed at HH24 to reduce hemodynamic load. Quantification of optical mapping from high-speed imaging of a voltage-sensitive dye [92] revealed an early emergence of an apex-first pattern in OTB hearts versus controls [35]. In contrast, LAL hearts had a delayed emergence of apex-first mature activation pattern at HH36, as not all hearts exhibit the apex-first activation pattern. The study showed that hemodynamic conditions influence maturation of cardiac activation patterns.

## 6. Conclusions and Perspectives for Future Studies

Results from various animal models show that blood flow is not only essential for heart development [8], but also hemodynamic stresses exerted by blood flow on cardiac walls must be within a certain range for the heart to grow normally and develop typical myocardial patterns [46]. Increased blood pressure drives myocardial growth, while increased volume drives dilation of the ventricles [46,48,71,72]. WSS also plays an important role on cardiac cushion development and subsequent formation of the valves [51,52,54], and heart septa [84]. The extent of hemodynamic stress deviation from normal dictates incidence of defect phenotypes [10].

While the molecular mechanisms by which hemodynamics affect development are being unraveled, more research is needed to fully understand and predict the linked interaction between blood flow and genetic processes that shape the morphology and function of the heart. New technological advances facilitate studying heart development and blood flow-induced responses in details more than ever before. However, accurate ways of computing hemodynamic stresses, such as CFD and FSI, and quantifying gene network interactions in response to hemodynamic stresses must be further developed.

After the fetal heart is fully formed, ventricular wall thickness and chamber size continue to be affected by blood flow, in particular pressure or volume overload conditions [38,39]. Several studies have shown that fetal myocardium responds to an increase in preload via increasing proliferation or hyperplasia [46,72]. Myocardial proliferation is significantly reduced after birth, when heart growth occurs mainly due to an increase in the volume of individual myocardial cells (hypertrophy). Therefore, the inventory of cardiac cells at the time of birth may be crucial for survival and subsequent quality of life [93]. These findings support the rationale for prenatal surgical interventions to mitigate certain types of CHDs [46]. For example, HLHS, characterized by an underdeveloped left heart, accounts for 25% of cardiac deaths within the first year of life [72]. Fetal aortic stenosis (a narrowing of the aorta) in HLHS promotes further underdevelopment. There have been successful reports on balloon dilatation for severe aortic stenosis as attempts to prevent left ventricular development arrest and its progression to HLHS [94]. Successful attempts at using maternal hyperoxygenation to improve blood flow in left-sided heart defects and proper development of heart and brain have also been reported [95]. These are cases in which prenatal intervention, aiming at restoring normal hemodynamics in the fetal heart, greatly improves and extends the quality of life of children with CHD. More research is needed to identify other cases in which early intervention could be beneficial, and to develop appropriate tools and strategies that allow for successful fetal interventions.

Early diagnosis can be critical to ensure prompt interventions to minimize cardiac tissue abnormalities [96,97,98]. Measurement of blood flow can be used as an early diagnostic tool and a starting point for planning fetal interventions. To save lives and improve quality of life in CHD, new technologies enabled by novel computational modeling for fetal blood flow, together with better understanding of the effects of hemodynamics on cardiac development, are needed to allow for early diagnosis, followed by early fetal intervention.

## Figures and Tables

**Table 1:** Hemodynamic consequences of surgical interventions in chicken embryos.

Hemodynamic Intervention	Stage Performed	Physiological Response
VVL	HH17-18	acute ↓Q → ↓stroke volume → ↓WSS
OTB	HH17-24	↓OFT diameter with ~ constant Q → ↓WSS ↑ventricular blood pressure → ↑contractile force → ↑σ
AL	HH21-24	↓Q in ventricle (and flow imbalance) → ↓stroke volume → ↓WSS

↓: decrease; →: leads to; ↑: increase.

## Data Availability

Not applicable.

## Abbreviations

**Grouping**	**Acronym**	**Term**
Cardiovascular Biology	RBC	red blood cells
	ECM	extracellular matrix
	OFT	outflow tract
	IVS	interventricular septum
	AVC	atrioventricular canal
	APS	aorticopulmonary septum
	PAA	pharyngeal arch artery
	Endo-MT	endocardial-to-mesenchymal transition
	EMT	epithelial-to-mesenchymal transition
	HH	Hamburger–Hamilton staging
Heart Conduction	HSP	His–Purkinje system
	AV	atrioventricular node
Congenital Heart Defects	CHD	congenital heart disease
	HLHS	hypoplastic left heart syndrome
	CoA	coarctation of the aorta
	d-TGA	d-transposition of the great arteries
	VSD	ventricular septal defect
	DORV	double outlet right ventricle
	APW	aorticopulmonary window
	TOF	tetralogy of Fallout
Fluid Dynamics	WSS	wall shear stress
	P	pressure
	ΔP	change in pressure
	Q	volume flow rate
	FSI	fluid structure interaction
	CFD	computational fluid dynamics
Hemodynamic Interventions	OTB	outflow tract banding
	VVL	vitelline vein ligation
	AL (LAL or RAL)	(left/right) atrial ligation
